# Investigating the relationship between emotional labor levels, job satisfaction, and burnout among nurse academicians: a structural equation modeling approach

**DOI:** 10.3389/fpsyg.2025.1677531

**Published:** 2025-12-03

**Authors:** Nadiye Barış Eren, Leyla Dinç

**Affiliations:** 1 Department of Nursing, Faculty of Health Sciences, Tarsus University, Mersin, Türkiye; 2 Department of Fundamentals of Nursing, Faculty of Nursing, Hacettepe University, Ankara, Türkiye

**Keywords:** academicians, burnout, emotional labor, job satisfaction, nursing

## Abstract

**Background:**

Nurse academicians interact face-to-face with nursing students during the teaching process and with individuals, families, and other healthcare professionals in their practice. This study examined the relationship between emotional labor levels, job satisfaction, and burnout among nurse academicians.

**Methods:**

This descriptive correlational study was conducted with 474 participants selected using a stratified sampling method among nursing academics working at public universities in seven geographical regions of Türkiye. Data were collected through the “Descriptive Characteristics Form,” the “Emotional Labor Scale,” the “Copenhagen Burnout Inventory,” and the “Minnesota Satisfaction Questionnaire.” Data were analyzed using descriptive statistics, the Kolmogorov–Smirnov test, skewness and kurtosis, *t*-tests, one-way ANOVA, the hierarchical regression model, and path analysis (a structural equation model).

**Results:**

Nurse academicians generally demonstrated genuine acting and experienced work-related burnout; however, they had high intrinsic job satisfaction. Surface acting and deep acting differed by gender, and we observed a significant relationship between deep acting, marital status, and duration of work. Emotional labor subscales were significant predictors of burnout subscales and job satisfaction. Specifically, surface acting showed a positive association with personal, work-related, and student-related burnout (β = 0.159, *p* < 0.001; β = 0.192, *p* < 0.001; β = 0.187, *p* < 0.001), while genuine acting was negatively associated with the same burnout subscales (β = -0.107, *p* = 0.026; β = -0.123, *p* = 0.010; β = -0.167, *p* < 0.001). Deep acting (β = 0.099, *p* = 0.010) and genuine acting (β = 0.103, *p* = 0.009) positively predicted job satisfaction.

**Conclusion:**

Deep acting and genuine acting had positive effects on nurse academicians’ job satisfaction, while surface acting increased burnout, genuine acting decreased burnout. Scientific activities are recommended for professional development to enhance the understanding of authentic and deep acting among nursing academics.

## Introduction

Globalization has increased the international mobility and trade of goods, services, and labor force, leading institutions and organizations to turn to cost-effective, productive, and customer-oriented strategies to survive in the competitive market economy environment. The importance of the service sector, which involves intensive human relationships, has been growing alongside countries’ economic development and progress. The service sector is labor-intensive, cannot be stored or transferred, is not concrete; does not produce a material product; involves simultaneous production and consumption; and relies on a mutual relationship between producers and consumers (Sayım and Aydın, 2011). Therefore, the performance and service quality of institutions and organizations are directly associated with customer satisfaction. Because customer satisfaction is affected by the attitudes and behaviors of service providers and workers, workers are expected to demonstrate professional attitudes and views in line with organizational goals and manage their emotions. Therefore, the service sector is a field in which emotional labor is experienced intensively.

Emotional labor was first mentioned in the book The Managed Heart: Commercialization of Human Feeling by the American sociologist Arlie Russell Hochschild. Hochschild (2012) defined emotional labor as managing feelings to produce publicly observable facial and bodily expressions that align with organizational goals. Hochschild defined two emotional labor subscales: surface acting and deep acting. Surface acting involves hiding real emotions and displaying emotions that are not actually felt. Deep acting focuses on the regulation of inner emotions to express desired emotions. In deep acting, the individual acts out or experiences the emotions expected from him or her by triggering emotions or using imagination. Ashforth and Humphrey (1993), who defined emotional labor as demonstrating expected emotions in the services provided by the organization, added genuine acting (the expression of genuine emotions) to these two subscales. Genuine acting refers to the real emotional expressions that workers experience naturally and demonstrate with less effort. For instance, the emotions of a nurse who sympathizes with a wounded child occur naturally.

Hochschild (2012) stated that surface acting caused emotive dissonance and triggered stress. Similarly, Ashforth and Humphrey (1993) reported that emotional labor increased good service expectations of service fields on one hand, and triggered emotive dissonance and self-alienation among workers on the other hand. Grandey (2000) defined emotional labor as the regulation of both emotions and behaviors to serve organizational goals, stating that emotional labor and burnout have a direct proportional relationship. According to Grandey, the main causes of this are that individuals experience conflicts between real emotions and the emotions that they reflect outwardly; this conflict causes tension, emotive dissonance, and work stress, and constant surface acting consumes emotional sources. By contrast, job satisfaction increases if workers’ natural emotions and the behaviors expected by the organization overlap (Lee and Ok, 2012). Chen et al. (2012) reported that surface acting is negatively associated with job satisfaction and positively associated with burnout, and deep acting is positively associated with job satisfaction and negatively associated with burnout.

Emotional labor is deeply experienced in academic activities that provide education training services requiring face-to-face interaction. According to the Turkish Law of Higher Education No. 2547 published in 1981, academic personnel must specialize in their field after their undergraduate education by pursuing postgraduate studies, conducting scientific research, publishing articles, participating in academic activities (congresses, symposiums, etc.), and educating and consulting for undergraduate, and postgraduate students. Therefore, in the process of face-to-face interaction, academicians experience emotional labor as learners and teachers. They can hide their true emotions through surface acting and emotion regulation (McRae and Gross, 2020). For instance, a teacher may feel angry at a student’s noncompliance and want to yell in frustration, but instead, they choose to redirect the student with a steady voice and calm face. As another example, a teacher may feel the urge to laugh out loud at a child’s humorous comment in class but instead adopt a stern expression and refocus the class on the instructional activity (Stark and Bettini, 2021). Through these strategies, teachers manage their emotions effectively and maintain a professional demeanor in the classroom (Waldbuesser et al., 2021).

Various studies in the literature on the emotional labor of academics indicated negative consequences of surface acting and positive effects of deep acting (Ozturk et al., 2015; Yücebalkan and Karasakal, 2016). For example, a study by Hao (2024) on the relationship between emotional labor and work performance among university teachers found that surface acting negatively affected academicians’ job performance, whereas deep acting enhanced their performance. A study by Lee and Vlack (2018) showed that surface acting caused negative emotions, such as anxiety and disappointment; by contrast, deep acting decreased anxiety among academicians by increasing positive emotions, including pleasure and pride. Zhang and Zhu (2008) showed that surface acting increased burnout and decreased satisfaction, whereas deep acting and genuine acting decreased burnout and increased satisfaction. Similarly, a study by Han et al. (2021) found that surface acting inhibited teaching efficacy and learning assessment, whereas deep acting and expressing natural emotions increased teaching efficacy in course design, teaching strategy, and learning assessment. Moreover, they investigated the antecedents of university teachers’ emotional labor strategies and found that teachers perceived the emotional job demands of teaching as facilitators for using surface and deep acting. By contrast, their perceived that instructional support decreased surface acting and increased their expression of naturally felt emotions. A study by Zheng et al. (2024) supported these findings; their sample of 316 university teachers in China preferred using deep acting and naturally felt emotions to convey authenticity, and this preference enhanced teaching efficacy in course design and assessment.

Health service is considered to be a labor-intensive service (Tatar Baykal and Ercan Türkmen, 2022). Nursing is the largest community in health services and is based on face-to-face communication with individuals and families who require care (Delgado et al., 2017). Because nursing is an applied discipline, nurse academicians must guide, consult, educate, and supervise students’ laboratory studies and clinical practices (Ozturk et al., 2015; Smith and Gray, 2001). Therefore, academicians’ roles and responsibilities require emotional labor in their interactions as academicians and educators. In addition, they must carry out emotional labor in their interactions with professionals from different health disciplines and patients who receive nursing services in the clinical education process. Hence, nurse educators experience emotional labor both in their academic lives and within the scope of their roles in the service sector. However, despite the difficulties of academic life, the level of emotional labor may vary because tenure, promotion, research, publication, and education activities also have a satisfying aspect. Although various studies have investigated emotional labor and the relationship between emotional labor, job satisfaction, and burnout, few studies have examined nurse academicians’ emotional labor and related factors (Ozturk et al., 2015). This study investigated the relationship between emotional labor levels, job satisfaction, and burnout among academicians working in nursing undergraduate programs using path analysis (the structural equation model). In this study, the following hypotheses were put forward and tested:

*H1:* Surface acting and genuine acting have an effect on the personal burnout, work-related burnout, and student-related burnout subscales of the burnout scale.

*H2:* Deep acting and genuine acting have an effect on job satisfaction.

*H3:* The personal burnout and work-related burnout subscales of the burnout scale have an effect on job satisfaction.

## Materials and methods

### Study design

This study applied a descriptive correlational design to investigate the relationship between emotional labor levels, job satisfaction, and burnout among academicians working in nursing undergraduate programs.

### Setting and participants

The target population was academicians working in undergraduate nursing programs at public universities in Turkey. The target population was calculated as 1,669 individuals using the data provided on the official website of the Higher Education Information Management System of Turkey on 25 January 2019.^1^ A similar study in Turkey (Ozturk et al., 2015) reported a minimum sample size of 312, calculated with a 95% confidence interval, a 0.05 margin of error, and a medium level of emotional labor. Considering this, the sample size of this study was calculated using a stratified sampling method according to the following formula: Strata weight = Number of units (sample size) ÷ Number of units in the target population (universe) = 312 ÷ 1,669 = 0.186.

However, as the structural equation model requires a medium sample size, the study recruited 474 individuals who volunteered to participate (Deliveli and Kiral, 2020). The number of academicians in the undergraduate nursing programs in the seven geographical regions of Turkey was multiplied by the calculated weight of the stratum, and the minimum sample size for that geographical region was calculated.

The study included academicians working in Nursing Faculties and the Nursing Departments of Health Sciences Faculties across Türkiye. Since, faculty members without a nursing background may also be employed in nursing education programs, academicians without a nursing degree were included as well.

The inclusion criteria were as follows: (1) being employed full-time or part-time as an academic staff member in a nursing education program, (2) having at least 1 year of academic experience, and (3) voluntarily agreeing to participate in the study.

Exclusion criteria were: (1) being on administrative, research, or sick leave during data collection, and (2) not actively involved in teaching or research activities related to nursing education.

### Instruments

Data were collected using the Descriptive Characteristics Form, the Emotional Labor Scale, the Copenhagen Burnout Inventory, and the Minnesota Satisfaction Questionnaire.

### The descriptive characteristics form

This form contains 10 questions regarding the sociodemographic information of academics, including their age, gender, marital status, number of children, education level, title, duration of working, working unit, and the field of undergraduate education.

### The Emotional Labor Scale

This study utilized the Emotional Labor Scale, developed by Diefendorff et al. (2005) and adapted to Turkish by Basım and Beğenirbaş (2012), to measure emotional labor. The scale is a five-point measurement tool composed of 13 items scored between 1 and 5. Its three subscales include surface acting (six items: 1, 2, 3, 4, 5, and 6), deep acting (four items: 7, 8, 9, and 10), and genuine acting (three items: 11, 12, and 13). The participant receives 1 point for “never,” 2 points for “rarely,” 3 points for “sometimes,” 4 points for “often,” and 5 points for “always.” Higher scores indicate an increased demonstration of emotional labor (Beğenirbaş and Basım, 2013). The Turkish reliability and validity study calculated a Cronbach’s alpha of 0.80 for the whole scale. The analysis results of the scale revealed a KMO value of 0.814 and the following secondary DFA values: AGFI: 0.878, GFI: 0.925, CFI: 0.945, and RMSEA: 0.079 (Basım and Beğenirbaş, 2012). This scale was suitable because it has few items, the statements are comprehensible, and it is user-friendly (Basım and Beğenirbaş, 2012; Diefendorff et al., 2005).

### The Copenhagen Burnout Inventory

The Copenhagen Burnout Inventory was developed by Kristensen et al. (2005) and adapted to Turkish by Bakoğlu Deliorman et al. (2009). The scale is composed of 19 items scored on a 5-point Likert scale, which are divided into three subsections: personal burnout (6 items: 9, 13, 14, 15, 17, and 19), work-related burnout (7 items: 2, 4, 6, 8, 10, 12, and 16) and client/service receiver (student)-related burnout (6 items: 1, 3, 5, 7, 11, and 18). The items on the scale receive 1 point for “never,” 2 points for “rarely,” 3 points for “sometimes,” 4 points for “frequently,” and 5 points for “always.” Scale scores are then recoded as 100 (always), 75, 50, 25, and 0 (never); hence, higher scores indicate higher burnout levels. All the burnout items are shown together, and the items on each subscale are related (Kristensen et al., 2005). The personal burnout subscale indicates the individual’s level of physical and psychological fatigue and weakness experienced. Work-related burnout indicates the level of perceived work-related physical and psychological fatigue and weakness. In this subscale, the question “Do you find the energy to spend enough time with your family and friends out of work time?” is reverse-scored. The client/service receiver-related burnout subscale indicates individuals’ perceptions of their physical and psychological fatigue and weakness in their relationships with clients (students). The Turkish reliability and validity analysis calculated a Cronbach’s alpha value of 0.92 for the whole scale. The KMO value was 0.944 (Bakoğlu Deliorman et al., 2009). This scale was chosen because it addresses different professions and measures personal burnout as well as work-related burnout and client/service receiver-related burnout. It can also be used for participants from different cultures (Bakoğlu Deliorman et al., 2009; Kristensen et al., 2005).

### Minnesota Satisfaction Questionnaire

Minnesota Satisfaction Questionnaire was developed by Weiss et al. (1967), and adapted to Turkish by Akkamış in 2010. The short form includes the subscales of intrinsic job satisfaction (12 items: 1, 2, 3, 4, 7, 8, 9, 10, 11, 15, 19, and 20) and extrinsic job satisfaction (8 items: 5, 6, 12, 13, 14, 16, 17, and 18). It contains 20 items scored on a 5-point Likert scale (Akkamış, 2010). A value of 1 indicates “I am not satisfied at all,” 2 indicates “I am not satisfied,” 3 indicates “I am not sure,” 4 indicates “I am satisfied,” and 5 indicates “I am very satisfied.” Scores on the scale range between 20 and 100, and higher scores indicate greater job satisfaction. Scores of 75 and above indicate high job satisfaction; scores between 26 and 74 indicate medium job satisfaction; and scores of 25 and below indicate low job satisfaction (Weiss et al., 1967). The intrinsic job satisfaction subscale comprises items related to the intrinsic nature of job satisfaction, including success, appreciation, the work itself, responsibility associated with the work, and change of duty as a result of promotion. The extrinsic job satisfaction subscale includes items related to organization policy and management, type of supervision, relationships with supervisors and colleagues, working conditions, and pay. The Turkish reliability and validity study reported a Cronbach’s alpha value of 0.83 for the whole scale (Akkamış, 2010). This scale was chosen because it is user-friendly and practical to use in diverse cultures (Akkamış, 2010; Weiss et al., 1967).

### Data collection

Data were collected between 1 May 2019 and 21 December 2019 from a total of 479 nurse academics working in nursing undergraduate programs of public universities in seven regions of Turkey. First, dates and times were determined for visiting the universities where the study was to be conducted. After official permission documents were obtained, the researcher visited these universities on the pre-scheduled dates, and collected the data through face-to-face meetings with the academicians. Since there were missing data in the forms of five participants, they were removed, and the data from the remaining 474 academicians were included in the analysis.

### Ethical considerations

The Noninvasive Clinical Studies Ethics Committee of the university approved the study protocol (Protocol No: 2019/09-15; 2 April 2019). Written permission was obtained from the Nursing Faculties and Health Sciences Faculties of the public universities where the study was to be conducted. Permission to use the scales was obtained through e-mail from the authors who performed the Turkish reliability and validity of the data collection tools. The academicians who agreed to participate in the study provided verbal and written informed consent.

### Data analysis

Data were analyzed using SPSS 22.0; numbers, percentages, means and standard deviations, medians, and minimum and maximum values were calculated. Normality analysis was performed using Kolmogorov–Smirnov analysis as well as skewness and kurtosis values. The reliability of the scales for this sample was assessed using Cronbach’s alpha. Differences in scores on the Emotional Labor Scale based on sociodemographic features were determined using an independent samples *t*-test for paired groups and one-way analysis of variance for three or more groups. The hierarchical regression model was utilized for predictive analysis. The model was reviewed in terms of multiple regression assumptions before analysis. The model met the assumptions in terms of linearity, multicollinearity, normality, homoscedasticity, autocorrelation, variance inflation factors value, and the condition index. Path analysis, a type of structural equation modeling, was used to examine the relationship between the scales, and the analysis was performed using the observable variables. These analyses utilized model fit values (χ^2^/df, GFI, AGFI, CFI, RMSEA, SRMR, IFI, PNFI, and PCFI) and standardized β coefficients, standard errors (SE), *t*-values, and *p*-values (Deliveli and Kiral, 2020). In the structural equation model, the Copenhagen Burnout Inventory was included with all three subscales (personal burnout, work-related burnout, and student-related burnout), and the Minnesota Satisfaction Questionnaire was included with the total job satisfaction score. The Emotional Labor Scale was included with the surface acting, deep acting, and genuine acting subscales. A value of *p* < 0.05 was considered statistically significant.

## Results

### Demographic characteristics of the participants

The average age of the participating academicians was 36.2 ± 7.8 years, and 57.4% were aged 35 years and below. Most participants were females (92.0%), and 67.9% were married. The average number of children was 0.8 ± 0.9, and 74.7% had 0–1 child. Most (67.3%) of the participants had a doctorate as their most recent education, and 42% worked as research assistants. The average working experience in their current institution was 7.7 ± 7.0 years, and 51.5% had been working in their institution for 6 years or more. The average total professional experience was 12.8 ± 8.7 years, and 52.5% had worked for 10 years or less (97.6%) (Table 1).

**TABLE 1:** Socio-demographic characteristics of the academicians (*n* = 474).

Features	*n*	%	$\bar{\text{X}}$ ± SD
**Age**			**36.2 ± 7.8**
Aged 35 years and below	272	57.4	
Aged 36 years and above	202	42.6	
**Gender**			
Woman	436	92.0	
Man	38	8.0	
**Marital status**			
Married	322	67.9	
Single	152	32.1	
**Number of children**			**0.8 ± 0.9**
0–1	354	74.7	
2–6	120	25.3	
**Education status**			
Undergraduate	10	2.1	
Postgraduate	145	30.6	
Doctorate	319	67.3	
**Title**			
Research assistant	199	42.0	
Lecturer	65	13.7	
Assistant professor	134	28.3	
Associate professor	45	9.5	
Professor	31	6.5	
**Years in current institution**			**7.7 ± 7.0**
5 years or less	230	48.5	
6 years or more	244	51.5	
**Total professional working time**			**12.8 ± 8.7**
10 years or less	249	52.5	
11 years or more	225	47.5	
**Working unit**			
Nursing faculty	259	54.6	
Health sciences faculty	215	45.4	
**Field of education**			
Nursing	462	97.6	
Midwifery	3	0.6	
Biology	3	0.6	
Faculty of veterinary medicine	3	0.6	
Other*	3	0.6	

*Medical biological sciences (1), faculty of medicine (1), chemical engineering (1).

### Data obtained from the Emotional Labor Scale, the Copenhagen Burnout Inventory, and the Minnesota Satisfaction Questionnaire

The mean score on the surface acting subscale of the Emotional Labor Scale was 1.84 ± 0.79 points, the mean score on the deep acting subscale was 2.89 ± 1.22 points, and the mean score on the genuine acting subscale was 4.38 ± 0.65 points. The Cronbach’s alpha coefficients of the scale and subscales (α = 0.84–0.91) revealed high reliability for this sample.

The mean scores on the Copenhagen Burnout Inventory subscales were as follows: personal burnout: 46.19 ± 18.4 points; work-related burnout: 48.32 ± 15.3 points; student-related burnout: 41.54 ± 17.5 points. The scores on each subscale followed a normal distribution. The personal burnout subscale showed high reliability for this sample (α = 0.90), but the other two subscales had medium reliability (α = 0.73–0.79).

The mean scores on the Minnesota Satisfaction Questionnaire showed that the mean scores were 46.32 ± 6.79 points for intrinsic job satisfaction, 27.94 ± 5.64 points for extrinsic job satisfaction, and 74.27 ± 11.4 points for total job satisfaction. The subscales of this scale were found to have high reliability (α = 0.85–0.91) and met the normal distribution assumptions (Table 2). Variables from the Copenhagen Burnout Inventory and the Minnesota Satisfaction Questionnaire were analyzed using the hierarchical regression model.

**TABLE 2:** Data obtained from the Emotional labor, the Copenhagen Burnout Inventory and the Minnesota Satisfaction Questionnaire.

Scales *vs.* subscales	$\bar{\text{X}}$ ± SD	Median	Min-max	Skewness	Kurtosis	Cronbach’s α
**Emotional labor**						
Surface acting	1.84 ± 0.79	1.66	1–5	0.985	1.092	0.84
Deep acting	2.89 ± 1.22	3.00	1–5	−0.119	−1.117	0.91
Genuine acting	4.38 ± 0.65	4.33	1–5	−1.102	1.069	0.84
**The Copenhagen Burnout Inventory**						
Personal burnout	46.19 ± 18.4	45.83	0.0–100.0	0.390	−0.354	0.90
Work-related burnout	48.32 ± 15.3	46.42	7.1–100.0	0.181	0.303	0.73
Student-related burnout	41.54 ± 17.5	41.66	0.0–100.0	0.256	0.218	0.79
**The Minnesota Satisfaction Questionnaire**						
Intrinsic job satisfaction	46.32 ± 6.79	47.00	22.0–60.0	−0.553	0.333	0.87
Extrinsic job satisfaction	27.94 ± 5.64	28.00	8.0–40.0	−0.637	0.783	0.85
Total job satisfaction	74.27 ± 11.4	76.00	31.0–100.0	−0.615	0.668	0.91

### Distribution of the average mean score of the emotional labor subscales according to sociodemographic variables

The participants’ emotional labor scores were similar across characteristics such as age, the number of children, education level, title, work experience in the profession, and working unit; no statistically significant differences were found (*p* > 0.05). The genuine acting subscale score distributions of female and male academicians were similar (*p* > 0.05), but male academicians had higher mean scores on the surface acting (2.12 ± 0.94) and deep acting (3.39 ± 1.15) subscales than female academicians (1.81 ± 0.77; 2.85 ± 1.22) (*p* < 0.05). The mean score on the deep acting subscale was higher for married academicians (2.98 ± 1.23) than for single academicians (2.70 ± 1.18) (*p* < 0.05). In addition, the mean score for deep acting was higher among academicians who had worked at their current institution for 6 years or more (3.00 ± 1.20) than among those who had worked at their current institution for 5 years or less (2.78 ± 1.23) (*p* < 0.05) (Table 3).

**TABLE 3:** Scores of the emotional labor subscales according to socio-demographic characteristics.

Socio-demographic characteristics	Surface acting	Deep acting	Genuine acting
	**$\bar{\text{X}}$ ± SD**	**$\bar{\text{X}}$ ± SD**	**$\bar{\text{X}}$ ± SD**
**Age**			
Aged 35 years and below	1.86 ± 0.81	2.90 ± 1.20	4.35 ± 0.66
Aged 36 years and above	1.80 ± 0.77	2.88 ± 1.24	4.42 ± 0.64
*t*-value	0.750	0.188	−1.077
*p-*value	*0.454*	*0.851*	*0.282*
**Gender**			
Woman	1.81 ± 0.77	2.85 ± 1.22	4.38 ± 0.66
Man	2.12 ± 0.94	3.39 ± 1.15	4.40 ± 0.51
*t*-value	−2.330	−2.631	−0.199
*p-*value	***0.020***	***0.009***	*0.843*
**Marital status**			
Married	1.84 ± 0.80	2.98 ± 1.23	4.37 ± 0.67
Single	1.82 ± 0.78	2.70 ± 1.18	4.39 ± 0.60
*t*-value	0.276	2.415	−0.262
*p-*value	*0.782*	***0.016***	*0.793*
**Number of children**			
0–1	1.84 ± 0.81	2.84 ± 1.20	4.39 ± 0.63
2–6	1.82 ± 0.74	3.05 ± 1.25	4.33 ± 0.70
*t*-value	0.223	−1.673	0.914
*p-*value	*0.824*	*0.095*	*0.361*
**Education status**			
Undergraduate + postgraduate	1.79 ± 0.83	2.83 ± 1.25	4.33 ± 0.73
Doctorate	1.86 ± 0.77	2.92 ± 1.20	4.40 ± 0.60
*t-*value	−0.873	−0.725	−1.109
*p-*value	*0.383*	*0.469*	*0.268*
**Title**			
Research assistant/lecturer	1.80 ± 0.83	2.85 ± 1.25	4.35 ± 0.67
Assistant professor	1.90 ± 0.75	2.88 ± 1.13	4.43 ± 0.58
Associate professor/professor	1.82 ± 0.70	3.05 ± 1.24	4.38 ± 0.67
*t*-value	0.714	0.745	0.516
*p-*value	*0.490*	*0.475*	*0.597*
**Years in current institution**			
5 years or less	1.80 ± 0.79	2.78 ± 1.23	4.37 ± 0.63
6 years or more	1.87 ± 0.79	3.00 ± 1.20	4.39 ± 0.66
*t*-value	−0.861	−2.004	−0.349
*p-*value	*0.390*	***0.046***	*0.727*
**Total professional working time**			
10 years or less	1.83 ± 0.78	2.89 ± 1.22	4.36 ± 0.63
11 years or more	1.84 ± 0.80	2.89 ± 1.22	4.40 ± 0.67
*t*-value	−0.222	−0.011	−0.529
*p-*value	*0.825*	*0.992*	*0.597*
**Working unit**			
Nursing faculty	1.90 ± 0.83	2.98 ± 1.20	4.34 ± 0.68
Health sciences faculty	1.76 ± 0.74	2.78 ± 1.21	4.43 ± 0.61
*t*–value	1.922	1.768	−1.450
*p-*value	*0.055*	*0.078*	*0.148*

Bold and italic values indicate statistically significant results (*p* < 0.05).

### Path analysis findings about the relationships between emotional labor levels, job satisfaction, and burnout levels

In the structural equation model, first the Burnout Inventory with its three subscales, the Minnesota Job Satisfaction Scale with its total job satisfaction score, and the Emotional Labor Scale with its three subscales were included in the analysis. The model posits that surface acting, deep acting, and genuine acting impact personal burnout, work-related burnout, and student-related burnout scales of the burnout scale, thereby affecting job satisfaction. The fit index values of the path analysis model of the relationship between emotional labor levels, job satisfaction, and burnout levels were χ^2^/df = 162.354; GFI = 0.658; AGFI = 0.595; CFI = 0.210; RMSEA = 0.584; SRMR = 0.261; IFI = 0.220; PNFI = 0.063; PCFI = 0.060. These values were considered as poor goodness-of-fit indices and the model was rejected because the proposed structural relationships did not adequately represent the observed data. When the model was examined, no significant relationship was found between the deep acting subscale and all subscales of the Burnout Inventory. Similarly, no relationship was found between surface acting and job satisfaction, or between student-burnout and job satisfaction. Therefore, theoretical and empirical considerations were used to refine the model. In light of this information, the unrelated paths were removed and the analysis was re-conducted. The paths from deep acting to the burnout subscales, from surface acting to job satisfaction, and from student-related burnout to job satisfaction were removed because these were not significant in the analyses. Previous studies on this topic have also shown weak relationships between deep acting and burnout (Ashforth and Humphrey, 1993; Grandey, 2000; Hochschild, 2012; Morris and Feldman, 1996), and between surface acting and job satisfaction, and that student-related burnout does not have a direct impact on job satisfaction (Orhan and Komşu, 2016; Özgür Güler and Veysikarani, 2019; Öztürk, 2020).

**FIGURE 1:**
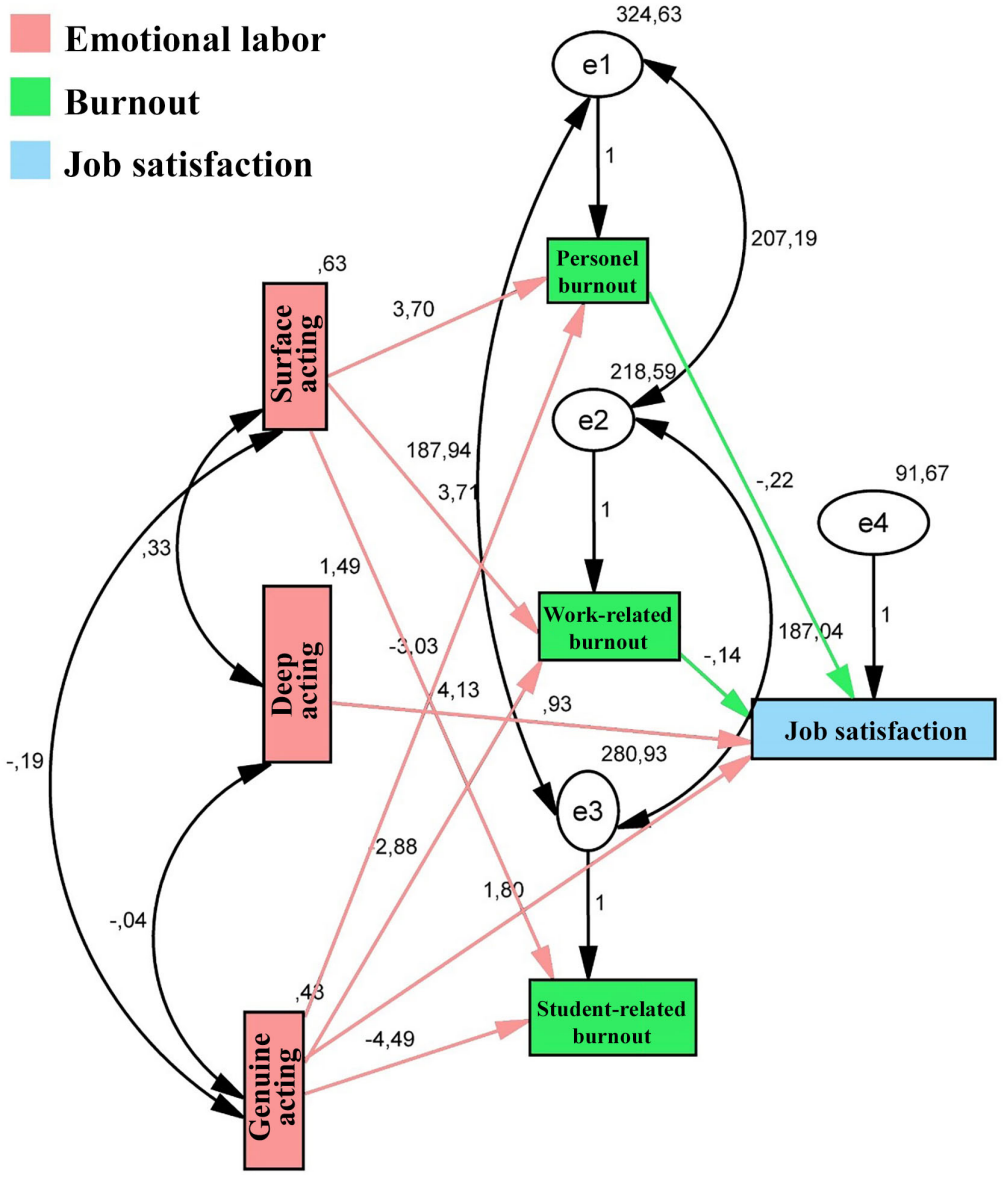
Path analysis between the emotional labor levels, job satisfaction, and burnout levels- modified model.

The new path analysis had fit index values of χ^2^/df = 0.871; GFI = 0.997; AGFI = 0.985; CFI = 0.918; RMSEA = 0.02; SRMR = 0.009; IFI = 0.985; PNFI = 0.537; PCFI = 0.538. These values are within the ranges of good fit and acceptable values. Table 4 presents the standardized β coefficients, standard errors (*SE*), *t*-values, and *p*-values among the variables of the developed model, and Figure 1 (the modified model) shows the path analysis between the emotional labor levels, job satisfaction, and burnout levels. The analysis results indicated a positive relationship between personal burnout (β = 0.159), work-related burnout (β = 0.192), and student-related burnout (β = 0.187) and surface acting and a negative relationship between these variables and genuine acting (β = -0.107, β = -0.123, and β = -0.167 respectively). A negative relationship was observed between personal burnout (β = -0.360) and work-related burnout (β = -0.187) and job satisfaction, whereas deep acting (β = 0.099) and genuine acting (β = 0.103) had a positive association with job satisfaction. The relationship between variables had a direct effect, and no indirect (mediating) effects were identified.

**TABLE 4:** Interaction between variables according to the path analysis.

Hypotheses	β	SE	*t-*value	*p-*value	Result
Personal burnout ← Surface acting	0.159	1.121	3.305	*<0.001*	Accepted
Work-related burnout ← Genuine acting	−0.123	1.118	−2.573	*0.010*	Accepted
Personal burnout ← Genuine acting	−0.107	1.363	−2.226	*0.026*	Accepted
Work-related burnout ← Surface acting	0.192	0.920	4.035	*<0.001*	Accepted
Student-related burnout ← Genuine acting	−0.167	1.268	−3.539	*<0.001*	Accepted
Student-related burnout ← Surface acting	0.187	1.043	3.956	*<0.001*	Accepted
Job satisfaction ← Personal burnout	−0.360	0.039	−5.753	*<0.001*	Accepted
Job satisfaction ← Work-related burnout	−0.187	0.047	−2.978	*0.003*	Accepted
Job satisfaction ← Deep acting	0.099	0.361	2.585	*0.010*	Accepted
Job satisfaction ← Genuine acting	0.103	0.687	2.623	*0.009*	Accepted

All *p* values are shown in italics and indicate statistical significance (*p* < 0.05).

## Discussion

Emotional labor in this study refers to the regulation of emotions to meet organizational expectations. It was operationalized through three subscales: surface acting (suppressing or faking unfelt emotions), deep acting (trying to genuinely feel the required emotions), and genuine acting (spontaneous expression of real emotions) (Ashforth and Humphrey, 1993; Grandey, 2000; Hochschild, 2012). The results showed that academicians generally experienced emotional labor in their professional lives and tended to display genuine emotions. These findings are consistent with previous research (Ozturk et al., 2015; Yücebalkan and Karasakal, 2016) but differ from others reporting higher levels of deep acting (Zhang and Zhu, 2008) or surface acting (Deliveli and Kiral, 2020).

Gender differences in emotional labor were also identified. Male academicians had higher mean scores on the surface acting and deep acting subscales than females. This finding contrasts with the theoretical expectations of Hochschild (2012), Grandey (2000), and Morris and Feldman (1996), who argued that females experience higher levels of emotional labor. However, while some studies found no gender differences (Deliveli and Kiral, 2020; Güler and Marşap, 2018), others support our results (Balcı et al., 2019; Yücebalkan and Karasakal, 2016). These findings suggest that male academicians may regulate their emotions more consciously in professional settings. In Turkish academia, such gender differences may be influenced by cultural norms that traditionally expect women to display empathy and nurturing behaviors, while men rely on more deliberate emotional control to meet institutional expectations (Grandey, 2000; Diefendorff et al., 2005). Additionally, emotional intelligence and supportive organizational climates can help reduce the negative effects of surface acting and enhance genuine emotional expression (Carmeli, 2003; Peña-Sarrionandia et al., 2015). Therefore, developing emotional intelligence and creating a positive institutional climate could help reduce burnout and emotional strain among academicians.

Marital status also influenced emotional labor. Married academicians had higher deep acting scores than single academicians. This may be because married individuals often take on multiple roles and responsibilities, requiring greater emotional regulation to maintain work–family balance. While some studies reported no significant relationship between marital status and emotional labor (Deliveli and Kiral, 2020; Yücebalkan and Karasakal, 2016), cultural expectations in Türkiye may explain this pattern. Marital roles often involve additional emotional and social responsibilities, encouraging deeper emotional regulation and more authentic emotional expression to meet both familial and institutional demands. Furthermore, emotional intelligence and supportive institutional climates can help individuals manage these dual expectations more effectively by promoting adaptive coping strategies and reducing burnout symptoms (Carmeli, 2003; Peña-Sarrionandia et al., 2015). Strengthening institutional support and fostering emotionally intelligent leadership may further promote balance between emotional labor and well-being among academicians.

Work experience was associated with emotional labor. Academicians with 6 or more years of experience scored higher on deep acting than those with 5 years or less. This finding aligns with previous studies showing that emotional labor tends to increase with professional experience (Ozturk et al., 2015; Yücebalkan and Karasakal, 2016). However, some research reported opposite results, including no relationship or even a negative association between experience and emotional labor (Balcı et al., 2019; Deliveli and Kiral, 2020; Güler and Marşap, 2018; Tunguz, 2016). The higher deep acting levels among more experienced academicians may reflect emotional adaptation. With professional maturity and exposure to complex interpersonal situations, emotional intelligence and self-regulation improve, leading to greater reliance on deep acting rather than surface acting (Carmeli, 2003; Peña-Sarrionandia et al., 2015). Supportive institutional climates that value collegiality and well-being can further sustain authentic emotional engagement and reduce burnout. Therefore, institutional policies that enhance emotional competence and provide mentoring for early career academicians may strengthen emotional resilience across academic settings.

Burnout in this study refers to physical and psychological exhaustion caused by prolonged stress. It was measured using three subscales: personal burnout (general fatigue), work-related burnout (exhaustion due to job demands), and student-related burnout (exhaustion from interactions with students) (Kristensen et al., 2005). The highest mean scores were observed on the work-related burnout subscale. This suggests that academicians experience higher levels of exhaustion primarily due to their professional responsibilities rather than personal or student-related factors. This finding supports previous research identifying workload and academic pressures as major contributors to burnout (Özen Kutanis and Karakiraz, 2013) but contrasts with studies reporting personal burnout as the dominant dimension (Karataş Aracı et al., 2016).

Job satisfaction in this study refers to the degree of contentment individuals feel toward their work. It was measured through two subscales: intrinsic satisfaction (achievement, recognition, and advancement) and extrinsic satisfaction (working conditions, pay, and interpersonal relations) (Weiss et al., 1967). The highest mean scores were found on the intrinsic job satisfaction subscale. This indicates that, despite high work-related burnout, academicians derive significant satisfaction from aspects inherent to their work—such as achievement, recognition, and academic autonomy. These results suggest that academicians perceive their profession not merely as a job but as a meaningful vocation and way of life (Dağdeviren et al., 2011; Özen Kutanis and Karakiraz, 2013; Yıldız et al., 2020).

This study found that emotional labor was an important variable affecting burnout and job satisfaction. We observed a positive relationship between the surface acting subscale of emotional labor and all subscales of the burnout scale; however, we found a negative relationship between genuine acting and these variables. Furthermore, no relationships were found between deep acting and burnout. Hence, whereas surface acting increased personal burnout, work-related burnout, and student-related burnout, genuine acting decreased burnout by reversing this effect on all subscales. The findings of this study align with the predictions of Hochschild (2012), Ashforth and Humphrey (1993), Grandey (2000), and Morris and Feldman (1996). Hochschild (2012), Ashforth and Humphrey (1993), and Grandey (2000), reported that expressing real emotions caused self-alienation among workers. Workers experienced emotive dissonance caused by surface acting (Hochschild, 2012), and this condition caused stress (Ashforth and Humphrey, 1993) and increased burnout (Grandey, 2000; Morris and Feldman, 1996). According to Hochschild (2012), this occurs when workers fail to manage their emotions successfully. A study involving academicians in Turkey reported that surface acting increased student-related burnout and work-related burnout, and genuine acting decreased personal burnout, student-related burnout, and work-related burnout (Yücebalkan and Karasakal, 2016). The findings of our study are in line with several other studies indicating that surface acting increases burnout (Bulutlar and Başkaya, 2015; Lee and Vlack, 2018; Zhang and Zhu, 2008) and genuine acting decreases burnout (Zhang and Zhu, 2008), but they do not align with other study results indicating that deep acting decreases burnout (Bulutlar and Başkaya, 2015; Zhang and Zhu, 2008) or increases burnout (Lee and Vlack, 2018). The non-significant relationship between deep acting and burnout observed in this study may be related to sample characteristics or cultural factors. In the cultural context of this study, individuals may perceive and regulate their emotions differently, which could have influenced how deep acting affects burnout. Emotive dissonance and the resulting increased burnout levels are somewhat expected in academicians demonstrating surface acting, as they suppress their real feelings and act as if they experience feelings that they are not (Morris and Feldman, 1996). In addition, surface acting could cause an increase in negative emotions, including anxiety and disappointment (Lee and Vlack, 2018) and lead to personal burnout, work-related burnout, and student-related burnout.

Regarding the effect of emotional labor on job satisfaction, deep acting and genuine acting had positive effects on job satisfaction, and there were no associations between surface acting and job satisfaction. Therefore, deep acting and genuine acting were found to increase job satisfaction. These findings align with the studies conducted by Hochschild (2012) and Ashforth and Humphrey (1993). Hochschild (2012) reported that deep acting resulting from the successful regulation of emotions increased workers’ job satisfaction. Ashforth and Humphrey (1993) reported that genuine acting and one’s freedom to express himself/herself increased personal well-being. Hence, job satisfaction, a component of personal well-being (Grandey, 2000), seems to increase with genuine acting. The findings of our study support other study results indicating that deep acting (Bulutlar and Başkaya, 2015; Zhang and Zhu, 2008) and genuine acting (Zhang and Zhu, 2008), increase job satisfaction. In our study, the reason for the increase in job satisfaction among academicians who demonstrate deep acting and genuine acting may be related to the fact that academicians get more satisfaction from their work by portraying the desired emotion or displaying spontaneous emotions. In addition, deep acting is thought to increase academicians’ job satisfaction by increasing positive emotions such as pleasure and pride and decreasing negative emotions such as anxiety (Lee and Vlack, 2018).

The personal burnout and work-related burnout subscales of the burnout scale had negative effects on job satisfaction. This suggests that personal burnout and work-related burnout decrease job satisfaction. This finding aligns with the results of other studies, which have reported a negative relationship between burnout and job satisfaction (Arslan and Acar, 2013; Özgür Güler and Veysikarani, 2019; Öztürk, 2020).

These findings also have broader international relevance. Studies across various countries have shown that surface acting increases burnout and decreases job satisfaction, while deep acting and the expression of genuine emotions enhance satisfaction and teaching performance (Zhang and Zhu, 2008; Han et al., 2021; Zheng et al., 2024). Similarly, deep acting has been linked to positive emotions and reduced anxiety among academicians (Lee and Vlack, 2018), and recent research confirmed that it improves job performance (Hao, 2024). Meta-analytic evidence further emphasizes that deep acting supports emotional well-being in public service professions (Humphrey, 2023). Moreover, emotional intelligence and supportive institutional climates can buffer the negative outcomes of surface acting and promote authentic emotional expression (Carmeli, 2003; Lee, 2017). Therefore, strengthening emotional intelligence and fostering supportive work environments may improve academicians’ job satisfaction and emotional well-being globally.

### Limitations and recommendations for future research

In this study, the use of structural equation model in examining the relationship between emotional labor, job satisfaction and burnout levels of nurse academicians is important in terms of revealing more statistically accurate and meaningful results. The fact that the study was conducted with academics working across all seven geographical regions of Türkiye is also a strong aspect in terms of the generalizability of the study results. For this reason, it contributes to the knowledge load of the literature on the subject. However, the results of this study encourage further cross-cultural research examining the relationship between emotional labor and various factors in the context of social and economic conditions of different countries that specifically focus on increasing job satisfaction and reducing burnout of nurse educators. In addition, we recommend planning interventions to raise academicians’ awareness of the concept of emotional labor.

However, this study has several limitations. First, all data were obtained through self-report instruments, which may have introduced response or social desirability bias. Second, data were collected over 8 months in 2019 using a descriptive correlational methodology based on structural equation modeling. Because a number of factors, particularly working conditions over time, can influence emotional labor, job satisfaction, and burnout, causal relationships cannot be derived from this study. Third, the sample consisted predominantly of female academics, reflecting the gender distribution in nursing academia in Türkiye, which may limit the generalizability of gender-based comparisons. Finally, because the study was conducted within the cultural and institutional context of Türkiye, the findings need to be evaluated and validated within the cultural structures and working conditions of different countries. Future studies with prospective design or mixed method approaches in different cultural contexts is recommended to confirm the findings of this study and to examine whether emotional intelligence or the institutional climate moderates the relationship between emotional labor and burnout, as supportive environments may buffer the negative effects of surface acting. Additionally, the findings highlight the importance of developing professional development programs and training aimed at improving emotional regulation skills among nurse academicians, which could enhance job satisfaction and reduce burnout.

## Conclusion

This study found that academicians working in nursing undergraduate programs most often demonstrated genuine acting, followed by deep acting and surface acting. Although we observed a positive relationship between surface acting and all subscales of the burnout scale, the genuine acting subscale was negatively related to personal burnout, work-related burnout, and student-related burnout. We observed a positive relationship between the deep acting and genuine acting subscales of the Emotional Labor Scale and job satisfaction. However, personal burnout and student-related burnout were negatively related to job satisfaction.

## Data Availability

The datasets presented in this article are not readily available because participants’ identities may be revealed. Requests to access the datasets should be directed to the corresponding author.

## Footnotes

1
https://istatistik.yok.gov.tr/
